# Cepharanthine hydrochloride reverses the mdr1 (P-glycoprotein)-mediated esophageal squamous cell carcinoma cell cisplatin resistance through JNK and p53 signals

**DOI:** 10.18632/oncotarget.22676

**Published:** 2017-11-27

**Authors:** Pengjun Zhou, Rong Zhang, Ying Wang, Dandan Xu, Li Zhang, Jinhong Qin, Guifeng Su, Yue Feng, Hongce Chen, Siyuan You, Wen Rui, Huizhong Liu, Suhong Chen, Hongyuan Chen, Yifei Wang

**Affiliations:** ^1^ Guangzhou Jinan Biomedicine Research and Development Center, Guangdong Provincial Key Laboratory of Bioengineering Medicine, College of Life Science and Technology, Jinan University, Guangzhou 510632, Guangdong, P. R. China; ^2^ Department of Pathogen Biology and Immunology, School of Basic Course, Guangdong Pharmaceutical University, Guangzhou 510006, Guangdong, P. R. China; ^3^ State Key Laboratory of Oncology in South China and Collaborative Innovation Center for Cancer Medicine, Sun Yat-sen University Cancer Center, Guangzhou 510060, Guangdong, P. R. China; ^4^ Guangdong Food and Drug Vocational College, Guangzhou 510520, Guangdong, P. R. China; ^5^ Guangzhou Institute of Pediatrics, Guangzhou Women and Children’s Medical Center, Guangzhou Medical University, Guangzhou 510623, Guangdong, P. R. China; ^6^ Department of Hepatobiliary Surgery, Xijing Hospital, Fourth Military Medical University, Xi’an 710032, Shanxi, P. R. China; ^7^ Guangdong Provincial Engineering Center of Topical Precise Drug Delivery System, Guangdong Pharmaceutical University, Guangzhou 510006, Guangdong, P. R. China

**Keywords:** cepharanthine hydrochloride, multi-drug resistance, MDR1, p-53, c-Jun/JNK

## Abstract

Esophageal squamous cell carcinoma (ESCC) is an aggressive malignancy that is often resistant to therapy. Nowadays, chemotherapy is still one of the main methods for the treatment of ESCC. However, the multidrug resistance (MDR)-mediated chemotherapy resistance is one of the leading causes of death. Exploring agents able to reverse MDR, which thereby increase the sensitivity with clinical first-line chemotherapy drugs, could significantly improve cancer treatment. Cepharanthine hydrochloride (CEH) has the ability to reverse the MDR in ESCC and the mechanism involved have not been reported. The aim of the study was to investigate the potential of CEH to sensitize chemotherapeutic drugs in ESCC and explore the underlying mechanisms by *in vitro* and *in vivo* studies. Our data demonstrated that CEH significantly inhibited ESCC cell proliferation in a dose-dependent manner, induced G2/M phase cell cycle arrest and apoptosis, and increased the sensitivity of cell lines resistant to cisplatin (cDDP). Mechanistically, CEH inhibited ESCC cell growth and induced apoptosis through activation of c-Jun, thereby inhibiting the expression of P-gp, and enhancing p21 expression via activation of the p53 signaling pathway. In this study, we observed that growth of xenograft tumors derived from ESCC cell lines in nude mice was also significantly inhibited by combination therapy. To our knowledge, we demonstrate for the first time that CEH is a potentially effective MDR reversal agent for ESCC, based on downregulation of the mRNA expression of MDR1 and P-gp. Together, these results reveal emphasize CEH putative role as a resistance reversal agent for ESCC.

## INTRODUCTION

Esophageal cancer (EC) is the fourth most common cancer in China, with a total of 477,900 new cases and 375,000 deaths projected to occur in 2015 [1]. Esophageal squamous cell carcinoma (ESCC) is one of the main histological types of EC in China with diverse cancer risk profiles [2]. ESCC accounts for over 90% of esophageal cancer cases and 5-year survival rates over the past 30 years is less than 20% [3-5]. At present, the clinical approach to ESCC is surgical treatment combined with radiotherapy and chemotherapy [6]. The most common treatment regimen for ESCC is the combination of cisplatin (cDDP) and 5-Fluorouracil (5-Fu) [7]. However, the obtained chemotherapy results were often barely satisfactory, mainly due to multiple drug resistance (MDR) [8, 9].

Once tumor cells are resistant to a single antitumor agent, the phenomenon of MDR confers upon cells the ability against many structurally unrelated antitumor agents [10-12]. Hence, the ability of cancer cells to acquire MDR is a major challenge to successful chemotherapy in a wide variety of advanced malignancies. One known cause of MDR is the over-expression of the ATP-binding cassette (ABC) transporters on the membranes of cancer cells. ABC transporters mediate an energy-dependent efflux can significantly decreasing the probability of successful treatment [13]. P-glycoprotein (P-gp), a membrane-associated glycoprotein which affiliated with the ABC superfamily, strongly linked to the MDR to play a role in drug efflux to reduce the drugs therapeutic effect [14-16]. An effective method to reverse P-gp mediated MDR is through its inhibitors to reduce the efflux of chemotherapeutic agents for increasing the sensitivity of tumor cells to chemotherapeutic drugs, therefore, to find and develop chemosensitizers is vital for MDR [17-20]. Although some compounds have been found as the candidate agents for MDR reversal, most of them exhibit pronounced toxic side effects resulting in their limited clinical application [21]. Compounds of natural sources have become the new trend in P-gp inhibitor discovery because they have less toxicity and higher effects [22].

**Figure 1:**
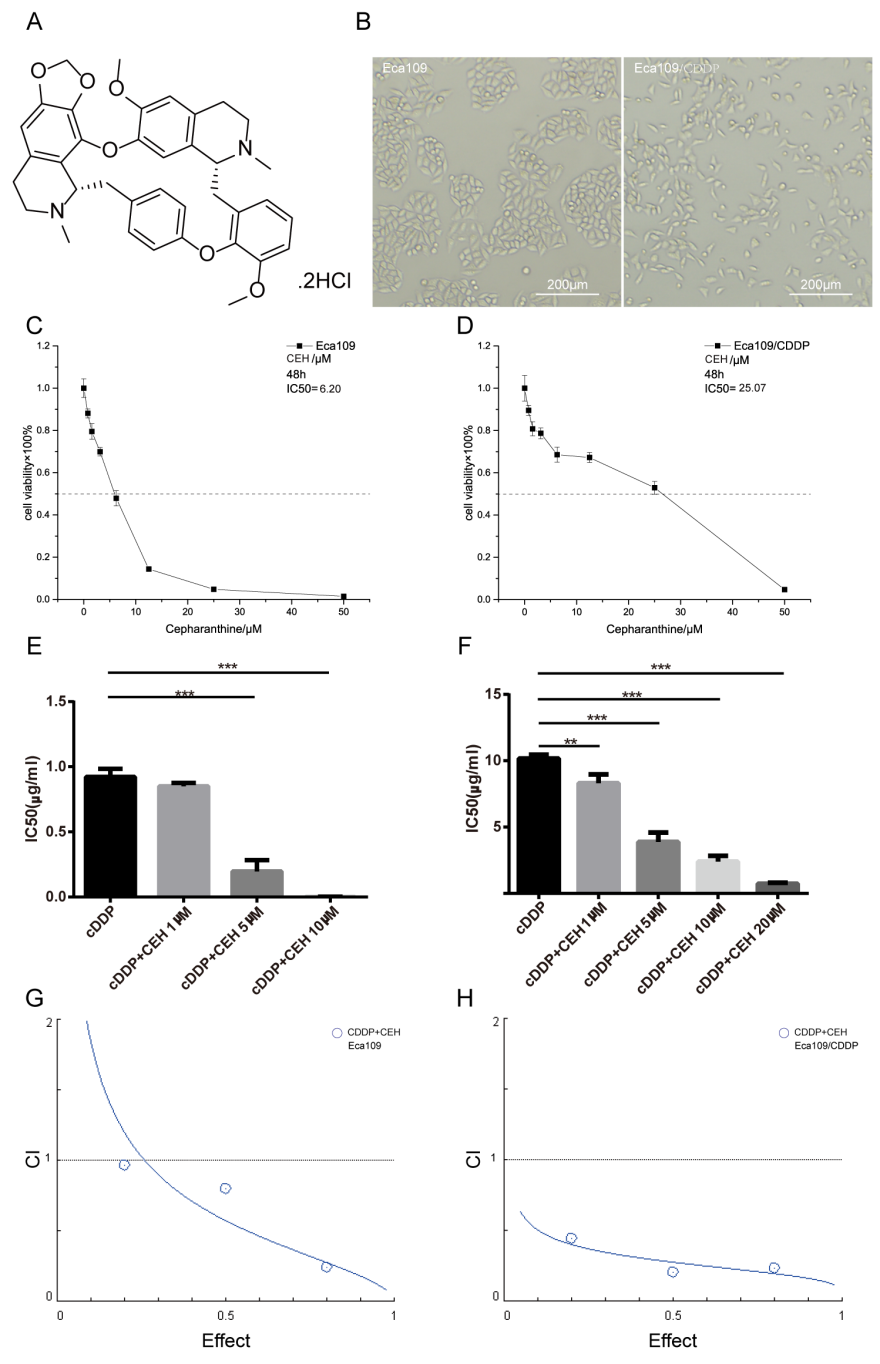
Inhibition of proliferation in esophageal cancer cell lines by cepharanthine hydrochloride (CEH) **(A)** Chemical structure of CEH used in the present study. **(B)** Morphological changes of Eca109 cells undergoing cisplatin induced drug-resistance. **(C and D)** Effects of CEH on the growth of the esophageal squamous cell carcinoma cell lines, Eca109 and Eca109/CDDP. Cells were treated with various concentrations of CEH for 48 h and cell viability was measured by MTT assay. **(E and F)** The effects of 48 h treatment with cisplatin (cDDP) and cDDP combined with various concentrations of CEH on the growth of Eca109 and Eca109/CDDP cells. The half maximal inhibitory concentration (IC_50_) was quantified. **(G and H)** The combination index (CI) of CEH and cDDP in Eca109 and Eca109/CDDP was below 1. All data are presented as the mean ± SD of three independent experiments. ^**^, P < 0.01; ^***^, P < 0.001 compared to the control.

Cepharanthine (CEP), a double-benzyl isoquinoline alkaloid monomer, which extracted from the plant *Stephania cepharantha Hayata* as an antitumor agent candidate for reversal of MDR [23-24]. Cepharanthine hydrochloride (CEH), a semi-synthetic derivative of CEP (Figure 1A), reverse MDR by inhibiting P-gp expression [25]. However, its antitumor effect and whether it can reverse MDR in ESCC remains largely unknown. In this paper, we investigated the effects of CEH combined with cDDP on the cell viability and apoptosis and explore the mechanisms on reversal of MDR potential for CEH *in vitro* and *in vivo*.

## RESULTS

### CEH increased the sensitivity of ESCC cells to cisplatin

First, we constructed a cisplatin-resistant cell line from the ESCC cell line Eca109, and named it Eca109/CDDP (Patent No. CN201511007006.2). Then, the cellular morphology of Eca109 and Eca109/CDDP was studied, we found that the morphology of Eca109/CDDP was irregular and misaligned, whereas that of Eca109 was fusiform and in alignment, and that the cell volume of Eca109/CDDP increased compared to that of Eca109 (Figure 1B). In order to verify the resistance of Eca109/CDDP cell lines, we performed the MTT assay after treated cisplatin for 48 hours, our data showed that the resistance index (RI) value of Eca109/CDDP was 11.21 ± 0.50, and in absence of cisplatin, Eca109/CDDP resistance to cisplatin was not affected (Supplementary Figure 1A-1C), this result demonstrated that Eca109/CDDP was a moderately resistant cell line. To evaluate the effect of CEH on cell viability in Eca109 and Eca109/CDDP, the MTT assay was performed 48 h after treatment. As shown in Figure 1C and Figure 1D, CEH significantly inhibited cell proliferation in a dose-dependent manner in ESCC cell lines, with IC_50_ values of 6.20 ± 0.17 μM in Eca109 and 25.07 ± 0.28 μM in Eca109/CDDP. We also found that under the CEH and cDDP combined treatment, the sensitivity of ESCC cells to cDDP significantly increased (Figure 1E and 1F, Supplementary Figure 1D-1F). Our results are also shown the combination index (CI) of CEH and cDDP in Eca109 and Eca109/CDDP was below 1 (Figure 1G and 1H), indicate that combination of cDDP and CEH showed synergistic effects.

Next, the MTT method was used to analyze the toxicity of CEH and cDDP in human normal somatic cells, i.e., human aortic vascular smooth muscle cells (HA-VSMC). As shown in Supplementary Figure 1G and 1H, the IC_50_ of CEH to HA-VSMC was significantly higher than that of ESCC. The reduced toxicity of CEH to normal cells suggested that it could be used as a potential agent for reversing ESCC cisplatin resistance.

### CEH induces cell cycle arrest and inhibition of cell proliferation in both Eca109 and Eca109/CDDP cell lines

**Figure 2:**
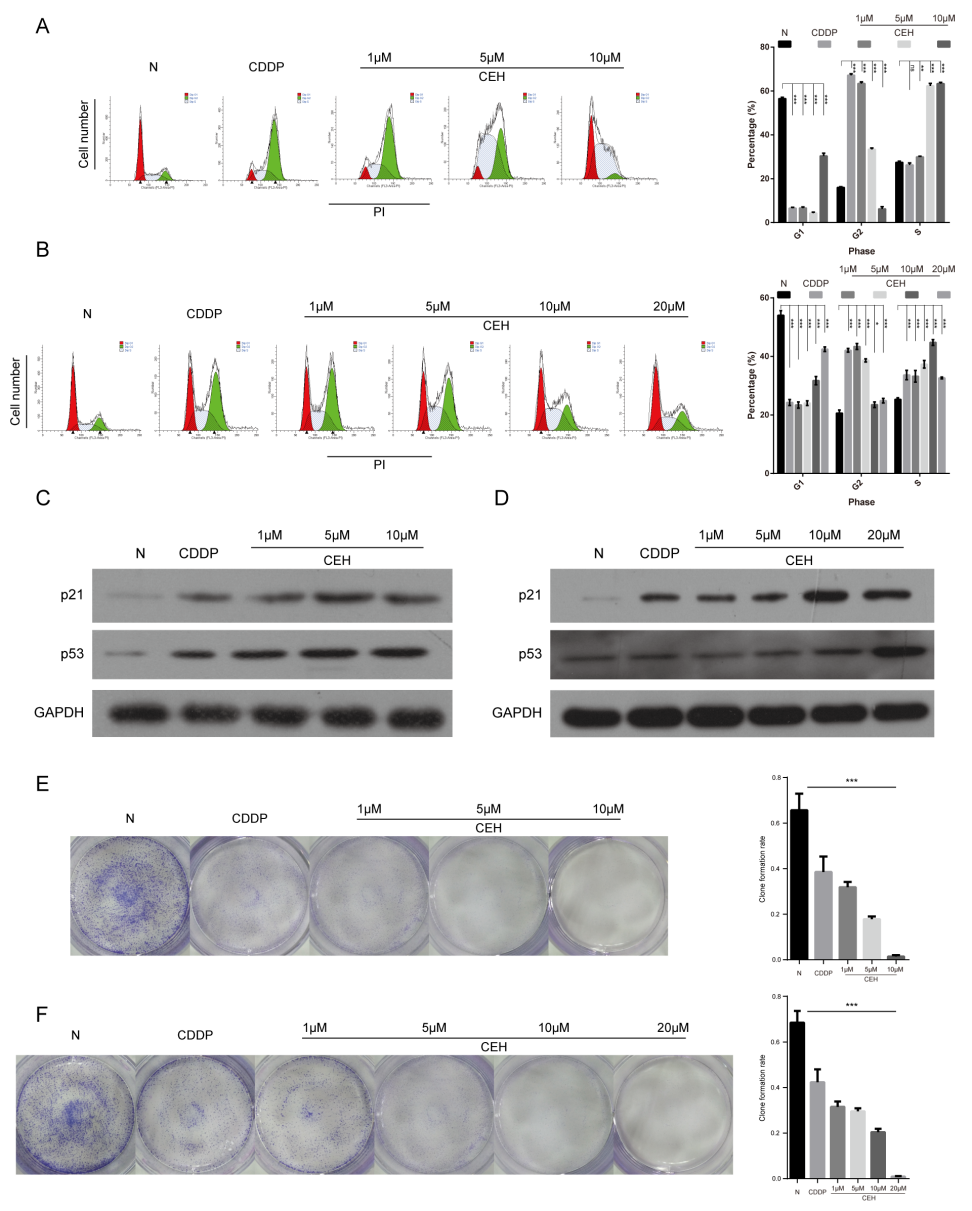
Induction of cell cycle arrest and inhibition of cell proliferation by cisplatin (cDDP) alone and combined with cepharanthine hydrochloride (CEH) in esophageal cancer cell lines **(A and B)** Cell cycle analysis. Percentages of Eca109 and Eca109/CDDP cells in the G1, S, and G2/M phases are presented respectively. Effects of cDDP and combined with various concentrations of CEH medication on cell cycle distribution. Eca109 (A) and Eca109/CDDP (B) cells were treated with 0, 1, 5, 10 and 20 μM CEH combined with cDDP for 48 h, and cell cycle distribution was measured by flow cytometry after PI staining. **(C and D)** p21 and p53 protein levels were determined by western blot analyses. GAPDH was used as the loading control. **(E and F)** Cells were treated with 0, 1, 5, 10 and 20 μM CEH combined with cDDP for 48 h; representative images of Eca109 (E) and Eca109/CDDP (F) clone formation are shown. ^***^, P < 0.001 compared with the control.

The inhibition of cell proliferation is associated with cell cycle, so we examined the effect of CEH combined with cDDP on the cell cycle progression in ESCC cells. As Figure 2A and 2B shown, compared with untreated control and the cDDP positive control, cell cycle was arrested at the G2/M phase when ESCC cells were treated with drugs for 48 hours. With the increase in CEH concentration, the cycle arrest showed dose-dependent. To explore the mechanism of cell cycle arrest, we investigated the cell cycle-related protein including p53 and p21. As shown in Figure 2C and 2D, CEH combined cDDP treatment with the different concentrations caused p53 and p21 expression increasing ESCC cell lines. Moreover, compared with untreated controls and the cDDP-treated cells, CEH and cDDP combined treatment inhibited cell proliferation in a dose-dependent manner in Eca109 and Eca109/CDDP (Figure 2E and 2F). These results illustrated that CEH, combined with cDDP, can induce ESCC cell cycle arrest and inhibit proliferation.

### CEH combined with cDDP increased apoptosis in Eca109 and Eca109/CDDP

**Figure 3:**
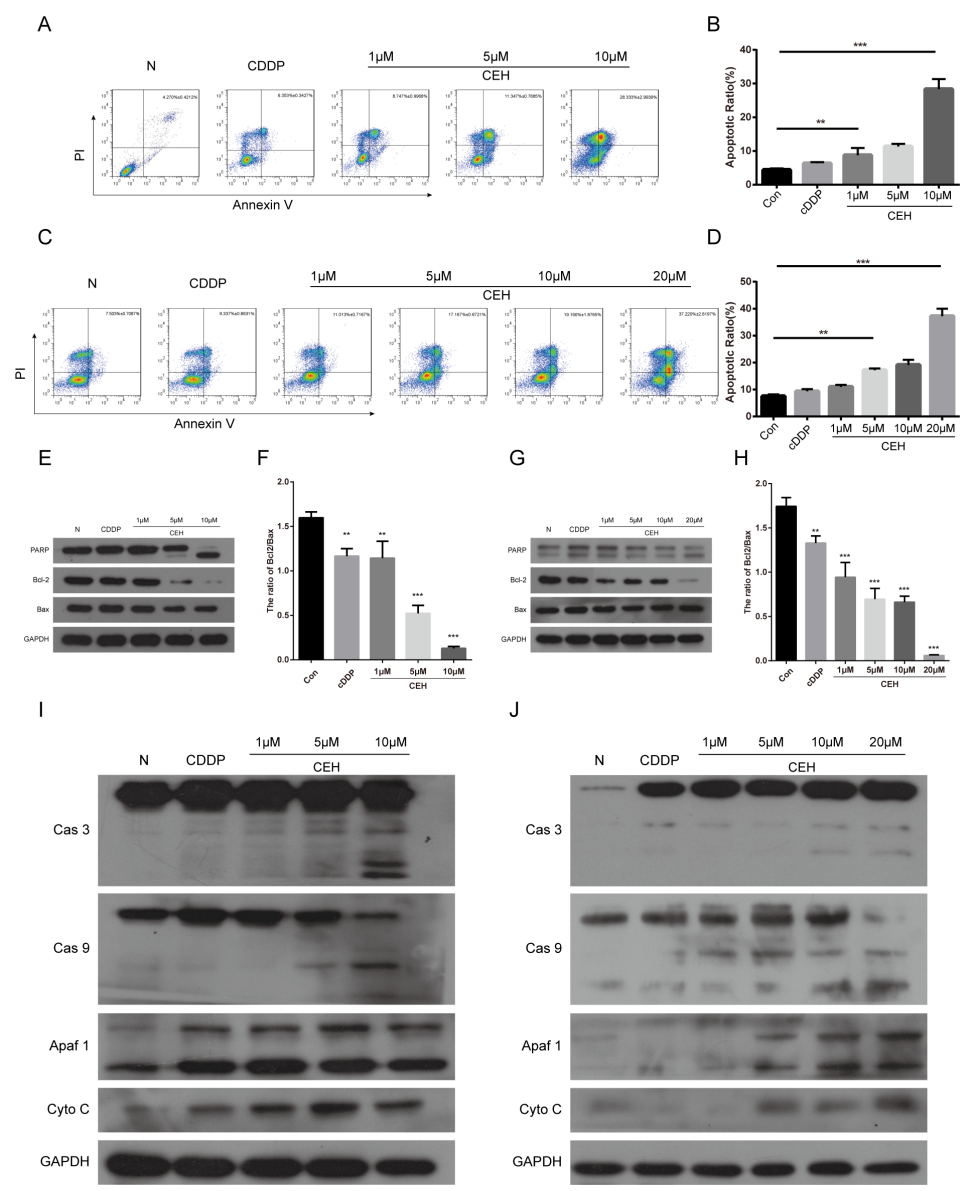
Induction of cell apoptosis by cepharanthine hydrochloride (CEH) in esophageal cancer cell lines **(A-D)** Induction of apoptosis by cisplatin (cDDP) and cDDP combined with various concentrations of CEH in Eca109 (A, B) and Eca109/CDDP (C, D) cells evaluated by Annexin-V-FITC/PI staining. **(E and F)** Western blot analysis of Bcl-2 family proteins and PARP. Eca109 cells were treated with cDDP and combined with various concentrations of CEH medication for 48 h. GAPDH was used as the loading control. **(G and H)** Western blot analysis of Bcl-2 family proteins and PARP. Eca109/CDDP cells were treated with cDDP and cDDP combined with various concentrations of CEH medication for 48 h. GAPDH was used as the loading control. Data are representative of three separate experiments. **(I and J)** Expression of caspases-3, caspases-9, Apaf1, and cytochrome c released from mitochondria was detected by Western blot analysis after treatment of Eca109 cells (I) and Eca109/CDDP (J) cells with cDDP and cDDP combined with various concentrations of CEH for 48 h. GAPDH was used as the loading control. Data are representative of three independent experiments. ^**^, P < 0.01; ^***^, P < 0.001; versus control.

Next, we examined the effect of CEH and its combination therapy on ESCC cell which were treated with cDDP alone or combination with CEH for 48 h and stained with Annexin V and PI. As shown in Figure 3A and 3C, treatment of CEH dose-dependent increased cDDP-induced cell apoptosis in ESCC cells. Then, we investigated the effects of CEH on apoptosis-related proteins. the results showed that CEH significantly increased PARP cleavage, but suppressed anti-apoptotic Bcl-2 expression, consistent with the observed upregulation of p53. However, Bax expression was not altered. (Figure 3E and 3G).

It is widely known that activation of the cytochrome c (Cyto-c)/caspase-9 pathway is one of the main signals transduction mediating apoptosis. Cyto-c, a necessary cofactor for apoptotic protease activating factor 1 (Apaf- 1) oligomerization and the subsequent activation of caspase-9 and -3 [26]. Therefore, we used western blot to detect the key protein of this signaling pathway. As shown in the Figure 3I and 3J, CEH combined with cDDP enhance the protein levels of the cleaved caspase-3 and -9 in both Eca109 and Eca109/CDDP cell lines in a dose-dependent manner. Moreover, the combination therapy significantly upregulated Apaf-1 as well as Cyto-c expressions. Taken together, our results indicate that CEH combined with cDDP could induce mitochondrial mediated apoptosis and caspase activation in Eca-109 and Eca109/CDDP cells.

### CEH dose-dependent reduction of P-gp mediated drug resistance in esophageal cancer cell and resistant cell line

**Figure 4:**
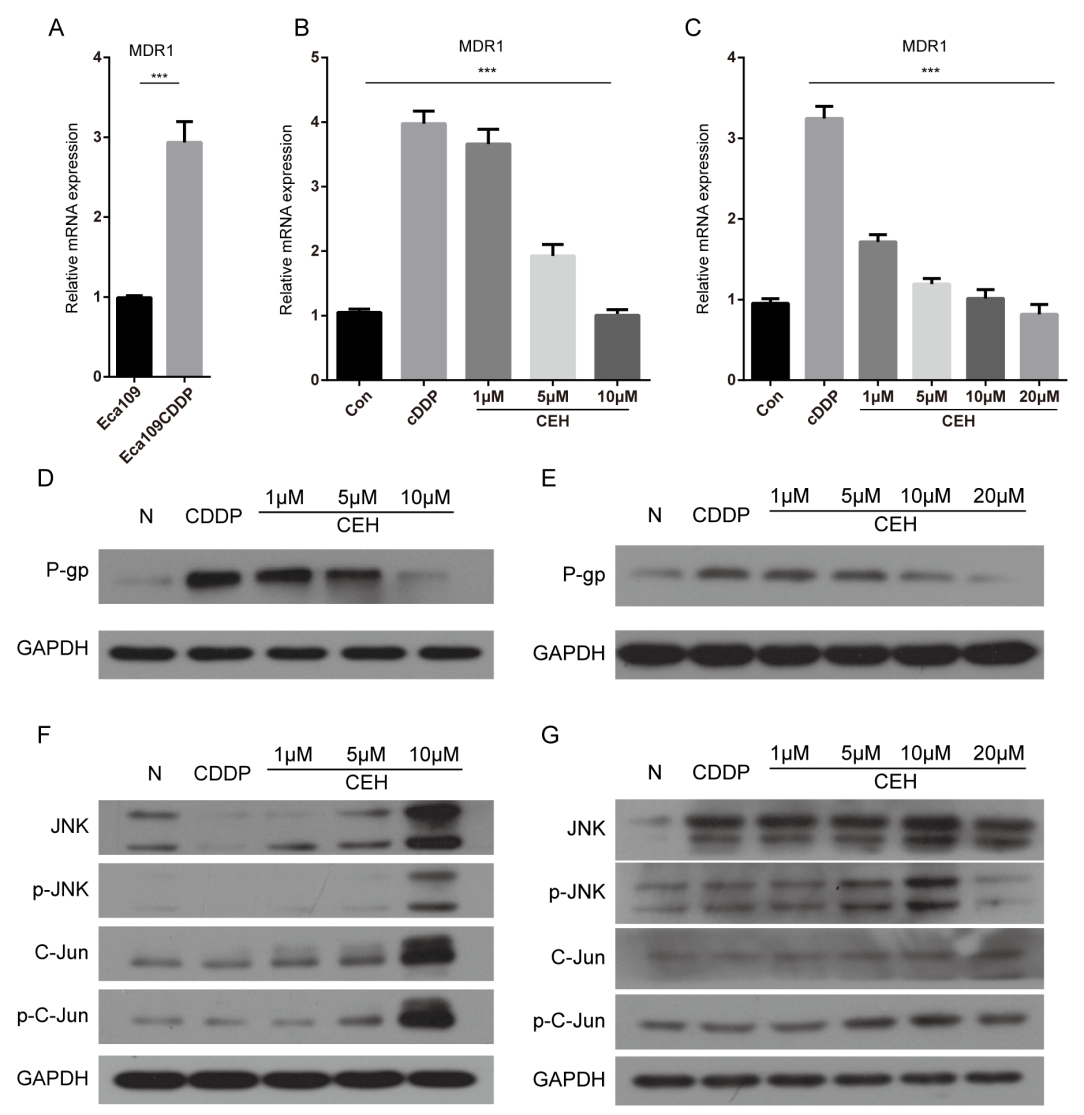
Cepharanthine hydrochloride (CEH) dose-dependent reduction of p-gp mediated drug resistance in esophageal cancer cell lines **(A)** Relative expression of *MDR1* mRNA was examined by qRT-PCR in Eca109 cells and Eca109/CDDP cells. Expression of the housekeeping gene GAPDH was used as reference. **(B and C)** Relative expression of *MDR1* mRNA was examined by qRT-PCR in Eca109 cells and Eca109/CDDP cells in the presence or absence of CEH under cisplatin (cDDP) conditions. Expression of the housekeeping gene GAPDH was used as reference. **(D and E)** Western blotting detection of P-gp protein expression in Eca109 and Eca109/CDDP cells treated with cDDP and cDDP combined with various concentrations of CEH for 48 h. GAPDH was used as the internal loading control. **(F and G)** Eca109 and Eca109/CDDP cells were treated for 48 h with cDDP and cDDP combined with various concentrations of CEH, western blotting detection of JNK, p-JNK, Jun and p-c-Jun proteins. GAPDH was used as an internal control. Data are presented as mean ± SD of three independent experiments. ^***^, P < 0.01 versus control.

It is well known that the expression of P-gp reduce intracellular drug concentration [27]. In order to verify the drug resistance mechanism of Eca109/CDDP, qRT-PCR and western blot analysis were used to quantify the *MDR1* mRNA and P-gp protein, respectively (Figure 4A-4E). Compared with its parental cell line Eca109, *MDR1* gene expression was significantly higher in the resistant cell line Eca109/CDDP. When both cell lines were treated with cDDP, the *MDR1* mRNA was significantly upregulated. These results suggested that the mechanism that confers cDDP-resistance to Eca109 cells involved upregulation of *MDR1* expression, leading to increased drug pumping and reducing the intracellular drug accumulation. With the increase in CEH concentration, *MDR1* expression levels were significantly reduced, and downregulation of P-gp also contributed to CEH-induced apoptosis (Figure 4B-4E), indicating that CEH could be used as MDR-mediated ESCC cisplatin resistance reversal agent.

Many experiments evidences shown that JNK, a member of the MAPK family, was closely related to the occurrence of MDR [28-31]. To explore the mechanisms of anti-tumor and resistance reversal activity of CEH, we determined the effects of CEH on the activities of c-Jun/JNK pathways. As shown in Figure 4F and 4G, CEH increase the expression and activation of c-Jun and JNK with a concentration dependent manner.

Mechanistically, CEH inhibited ESCC cell growth, induced apoptosis through repressing phosphorylation of c-Jun and reduced P-gp expression by the activation of c-Jun/JNK signaling cascades, which led to the reversal of P-gp-mediated cDDP resistance and promotion of mitochondrial-mediated apoptosis.

### JNK inhibitor SP600125 and p53 inhibitor PFTα can partially reversed apoptosis and cell cycle arrest due to cDDP and CEH combined treatment in Eca109 and Eca109/CDDP cells

**Figure 5:**
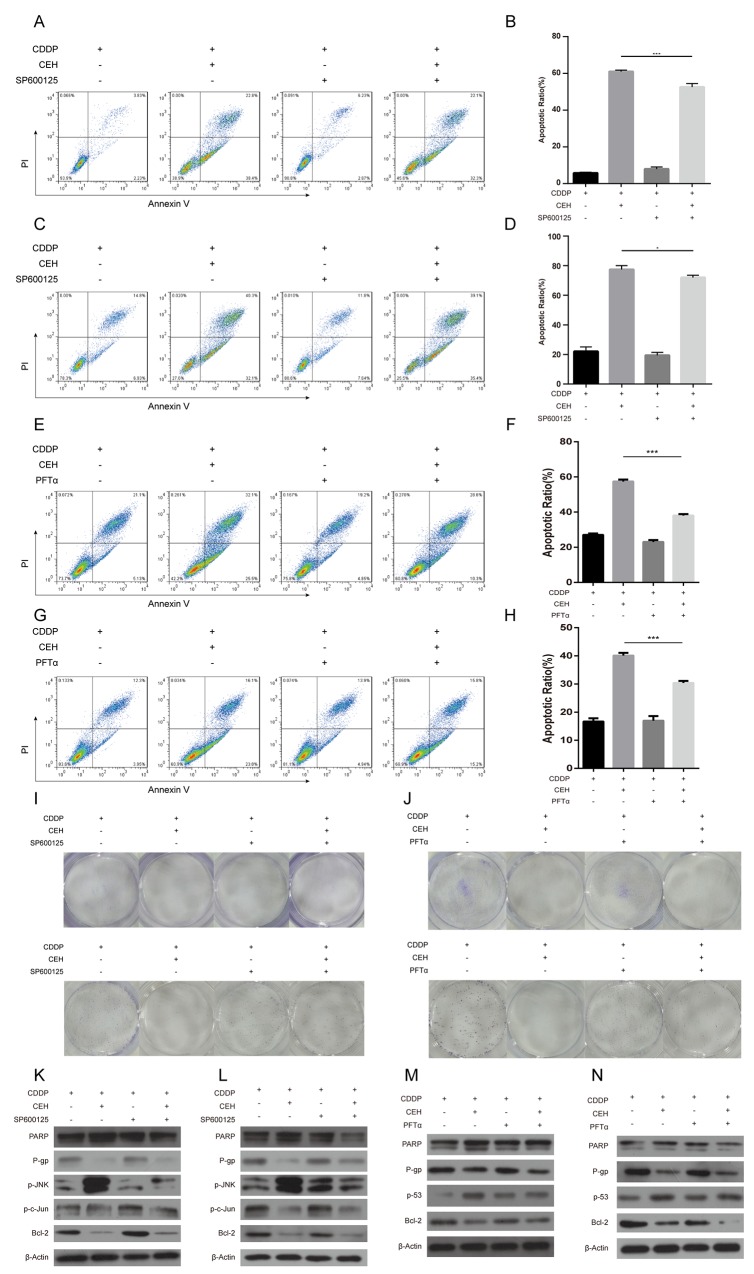
JNK inhibitor SP600125 and p53 inhibitor PFTα can partially reverse the apoptosis and cycle arrest induced by combined cisplatin (cDDP) and cepharanthine hydrochloride (CEH) treatment in Eca109 and Eca109/CDDP cells **(A-D)** Cells were treated with 5 μM CEH and 5 μM SP600125 alone or in combination, in addition to cDDP for 48 h; induction of apoptosis in Eca109 cells (A, B) and Eca109/CDDP (C, D) evaluated by Annexin-V-FITC/PI staining. **(E-H)** Cells were treated with 5 μM CEH and 0.5 μM PFTα alone or in combination, in addition to cDDP for 48 h; induction of apoptosis in Eca109 (E, F) and Eca109/CDDP (G, H) cells evaluated by Annexin-V-FITC/PI staining. **(I and J)** Cells were treated with 5 μM CEH, 5 μM SP600125 (I) or 0.5 μM PFTα (J) alone or in combination, in addition to cDDP for 48 h; representative images of Eca109 (upper panel) and Eca109/CDDP (lower panel) clone formation are shown. **(K-N)** Western blotting detection of PARP, P-gp, phosphorylated JNK (p-JNK), phosphorylated c-Jun (p-c-Jun) and Bcl-2 protein expression in Eca109 (K, M) and Eca109/CDDP (L, N) cells treated with 5 μM CEH, 5 μM SP600125 or 0.5 μM PFTα alone or in combination, in addition to cDDP for 48 h. β-Actin was used as an internal control. Data represent the mean ± SD of at least three independent experiments. Data are presented as mean ± SD. ^*^, P < 0.05; ^**^, P < 0.01; ^***^, P < 0.001.

We have previously demonstrated that CEH up-regulated the expression of P53 and JNK, Then, we used JNK inhibitor SP600125 or p53 inhibitor PFTα combined with CEH to determine the effects of CEH on the cDDP-induced expression of P-gp in Eca109 and Eca109/CDDP cells. As shown in Figure 5A-5D, tretment of SP600125 decreased CEH-induced cell apoptosis. Similarly, tretment of PFTα significantly decreased CEH-induced cell apoptosis (Figure 5E-5J). Furthermore, we found that the tretment of SP600125 sifnificantly reversed the P-gp expression inhibited by CEH (Figure 5K and 5L). In addition, treatment of PFTα significantly increased anti-apoptotic Bcl-2 expression, but decreased PARP cleavage (Figure 5M and 5N). Together, these findings suggest that CEH increases cell apoptosis and decareses the expressions of MDR1 mRNA and P-gp might be mediated by the activation of JNK and p53 pathways.

### CEH combined with cDDP inhibited ESCC xenograft tumor growth

**Figure 6:**
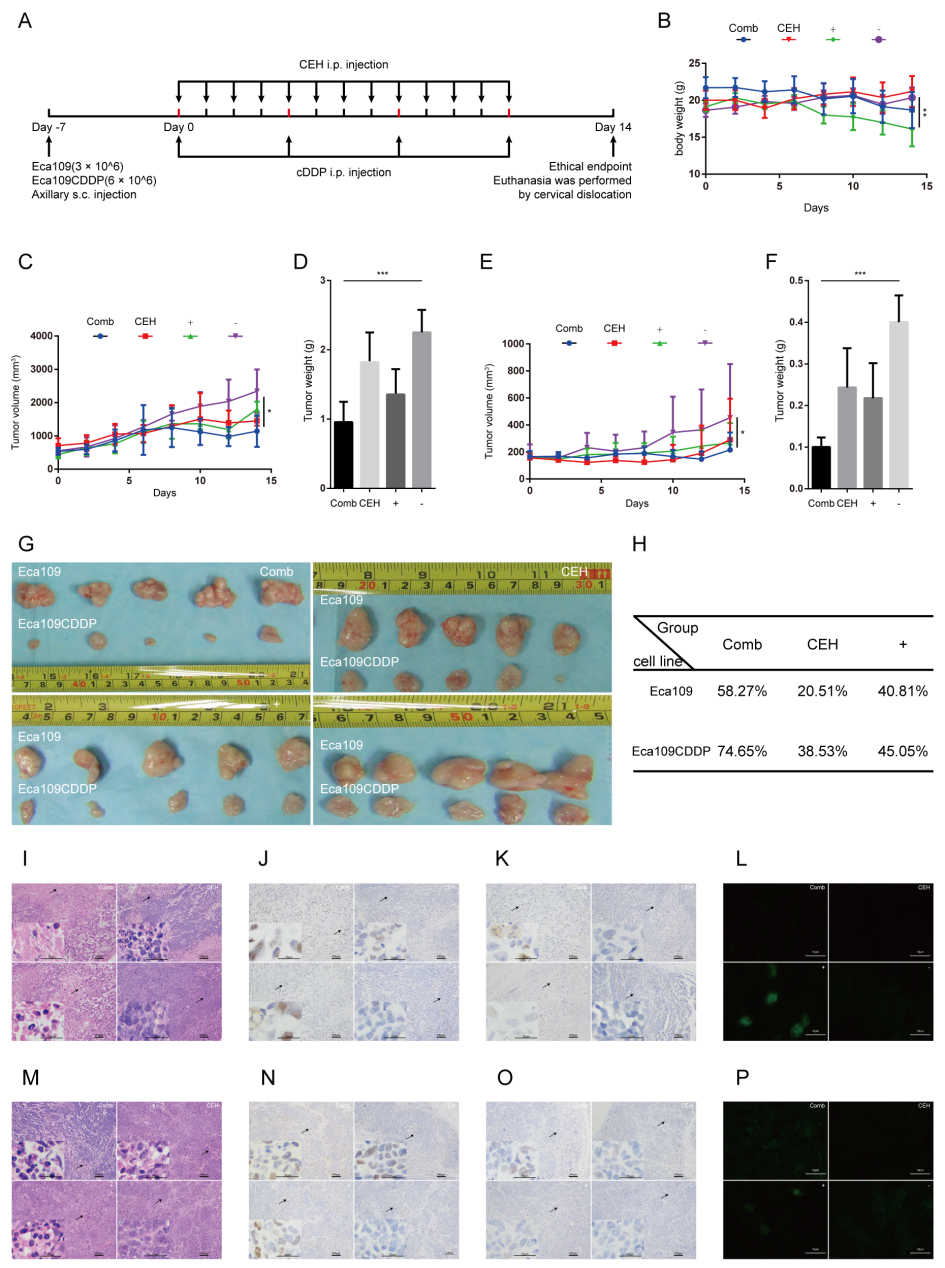
Cepharanthine hydrochloride (CEH) combined with cisplatin (cDDP) inhibits esophageal squamous cell carcinoma xenograft tumor growth **(A)** Time line of esophageal squamous cell carcinoma cell inoculation and drug treatment. **(B)** Time courses of animal weight. **(C)** Time courses of Eca109 cells xenograft tumor growth. **(D)** Bar graphs represent the mean weight of the Eca109 cells xenograft tumor after various treatments. **(E)** Time courses of Eca109/CDDP cells xenograft tumor growth were measured in each group at the indicated time point of various treatments. **(F)** Bar graphs represent the mean weight of the Eca109/CDDP xenograft tumor after various treatments. **(G)** Visual comparison of the dissected tumor tissues. Representative pictures of tumor growth in mice treated with vehicle control and the various treatments are shown. **(H)** Effects of CEH combined with cDDP on tumor inhibition rate of tumor mice. **(I and M)** Images are the representative H&E stained esophageal squamous cell carcinoma xenografts, originating from Eca109 cells (I) and Eca109/CDDP cells (M). (J and N). Expression of c-Jun in tumor tissues, originating from Eca109 cells **(J)** and Eca109/CDDP cells **(N)**, was assessed by immunostaining, scale bars: 25 μm. (K and O) Expression of phosphorylated c-Jun in tumor tissues, originating from Eca109 cells **(K)** and Eca109/CDDP cells **(O)**, was assessed by immunostaining, scale bars: 25 μm. (L and P) Expression of P-gp in tumor tissues, originating from Eca109 cells **(L)** and Eca109/CDDP cells **(P)**, was assessed by immunofluorescence, scale bars: 25 μm. Data are presented as mean ± SD. ^**^, P < 0.01; ^***^, P < 0.001. N = 5 in each group.

Next we inspected the effect of CEH on the growth of xenograft ESCC tumors. The experimental setup, including ESCC cell inoculation and drug treatment, is shown in Figure 6A. In the control group xenograft tumors grew faster than the group which treated with CEH, the tumor volume was significantly higher than CEH-treated group (Figure 6C, 6E, and 6G). However, the group treated with CEH did not affect body weight of nude mice compared with the control group, these results demonstrate that CEH does not affect the health life of the mice (Figure 6B). Compared with CEH and cDDP monotherapy groups, the combined therapy with CEH and cDDP activated the c-Jun/JNK signaling pathway in tumor-bearing mice and inhibited expression of P-gp (Figure 6I-6P). Our data suggested that CEH activated c-Jun/JNK pathway *in vivo*, and combined with CEH can significantly inhibit tumor growth compared with cDDP group (Figure 6H). Taken together, these data indicated that CEH could also effectively increase the anti-tumor effect of cDDP *in vivo*.

**Figure 7:**
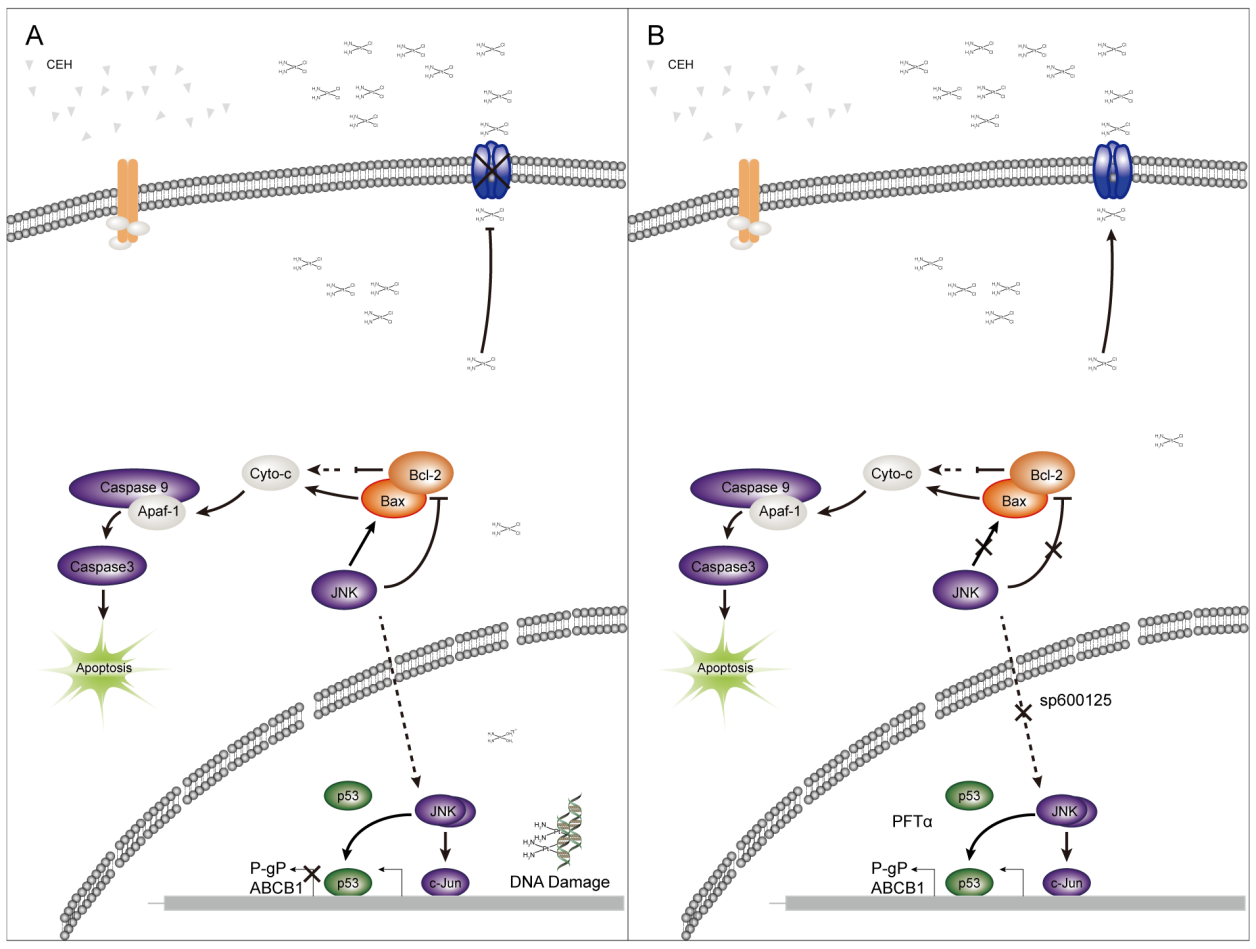
A simplified model of the mechanisms underlying antitumor effects of cepharanthine hydrochloride (CEH) combined with cisplatin in esophageal squamous cell carcinoma **(A)** CEH result in the reversal of MDR in ESCC through the activation of c-Jun/JNK and P53 signaling pathways. **(B)** Inhibitors of JNK and p53 can somewhat restore the reversion of MDR mediated by CEH.

This work clearly revealed that the combination therapy with cDDP and CEH had synergistic cytotoxic on ESCC, and the combination treatment stimulated apoptosis through downregulation of anti-apoptotic Bcl-2, upregulation of apoptotic Apaf-1, P21 and p53 expression, activation of the c-Jun/JNK signaling pathway, inhibition of P-gp expression, and reduction of the pumping of cDDP from ESCC cells to increase the accumulation of cDDP in tumor cells and increase tumor cells sensitivity to cDDP. Finally, CEH led to the reversal of MDR in ESCC via the activation of c-Jun/JNK signaling pathway (Figure 7), suggesting that CEH may be a novel drug for the treatment of clinical cancer chemotherapy in the future.

## DISCUSSION

In China, from 2003 to 2007, esophageal cancer was ranked the sixth most important cause of new cancer cases and the fourth primary cause of cancer-related deaths [32]. Although the treatment strategy for ESCC has been made, the prognosis going is still very slow [33]. The NCCN guidelines recommend combinational drugs to treat ESCC, such as cDDP combined with Dox and 5-Fu or 5-Fu alone. However, the most important problem related to ESCC therapy is the intrinsic resistance to chemotherapeutics [34]. As anticancer drugs are widely used, MDR is increasingly common with ESCC patients, which affects their treatment directly. Hence, profound discovery and understanding of its mode of action may lead to new therapy to overcome cisplatin-resistance and improve overall survival of patients with ESCC [35]. While research has made advances in the field of MDR, it remains a huge problem for clinical treatment [25] as more than 90% of all cancer deaths (more than 11 million by 2020) are considered to be associated with MDR [36, 37].

The mechanism of resistance to chemotherapy is very complex, one of the main mechanisms is dependent on P-gp-mediated increase efflux of drugs [38]. P-gp belongs to the ABC superfamily transporter which is the main drug efflux transporters associated with chemotherapy failure in cancer [39-42]. Much attention has been paid to the molecular mechanisms regulating the expression of these transporters as a viable approach to identify novel drug targets in circumventing MDR clinically. In our study, we constructed an *in vitro* MDR model and named it Eca109 / CDDP which had 12-fold more resistant to cDDP than the parental Eca109 cell line did. In this cell line the *MDR1* gene expression was significantly higher, therefore, we use it to study the role of CEH in MDR.

Small molecules extracted from Chinese herbal medicine were reported in literature to be able to reverse the MDR of malignant tumors [43-51]. CEP, a naturally active alkaloid compound, produces from *Stephania cepharantha Hayata*. Some reports have provided evidence that CEP has therapeutic potential for many diseases, and the side effects of CEP have rarely been reported [52-63]. In many biological activities of CEP, more attention has been paid to the increase of the sensitivity of tumor cells to chemotherapeutic drugs [64-68]. These findings suggested the potential of CEP to be a novel adjunct to chemotherapy. This sensitization mechanism might be that CEP restore the sensitivity of tumor cells to chemotherapeutic drugs by affecting the function of the cell membrane and thus lead to the accumulation of drugs in cancer cells [69, 70].

CEH (Figure 1A), manufactured by salification from CEP, was used in this study. Although CEH has been used as an antitumor agent and MDR-reversing agent in different types of cancers and one of the its identified MDR mechanisms include inhibition of P-gp expression and function, what its antitumor effect is and whether the MDR can be reversed in ESCC remains largely unknown. Therefore, we examined the potential activity of CEH in anti-tumor and reversal of drug resistance in this study. We found that CEH did not affect proliferation of normal cells up to a concentration of 6.25 μM; the IC50 value was 19.52 μM. Furthermore, we analyzed the combination of CEH and CDDP in synergies (CI < 1) by CalcuSyn software [71, 72], finding that the combination of CEH and CDDP met the sensitized and attenuated principle. Han et al. reported that CEH had a direct cytotoxic effect on human chronic myeloid leukemia cell line K562 and K562/ADR, a Dox-resistant cell line with stable MDR phenotype induced by Dox [25]. Similarly, we found that CEH could inhibit the growth of esophageal squamous cell lines Eca109 and Eca109/CDDP, and the antineoplastic activity showed concentration dependence.

Recent evidence indicates that JNK and NF-κB often regulate of *MDR1* expression [30, 73-74]. JNK regulates a series of cellular biological processes, including the expression of MDR1 gene, through c-Jun transmitted signals [30, 75]. Previous studies have reported that CEH on promote c-Jun expression and phosphorylation [76]. However, little was known how CEH reversal of MDR by regulating P-gp expression through the JNK/c-Jun signaling pathway, therefore, we focused JNK/c-Jun as a target to investigate the mechanism of CEH reversing MDR in ESCC cells.

To determine whether CEH reverse the expression of P-gp by activating JNK and reverse MDR in Eca109 and Eca109/CDDP cell line, we use JNK specific inhibitor SP600125 [77] to inhibit the activation of JNK Our results showed that CEH-mediated upregulation of MDR1 mRNA and P-gp was significantly reduced when using inhibitors of JNK. It demonstrated that CEH reversed MDR by activating the JNK signaling pathway of which induced *MDR1* mRNA and P-gp expression. Our results are consistent with previous reports by Sui et al. and Bark et al. [30, 78]. In addition, there are other signaling pathways involved in mediation of *MDR1*/P-gp in Eca109/CDDP cells. Therefore, we next investigated other signaling molecules implicated in the regulation of CEH-mediated reversal of MDR.

Inactivation of the p53 tumor suppressor gene occurs in over half of all human tumors, implying that loss of this gene represents a fundamentally important step in the pathogenesis of cancer [79]. p53 might cause cell cycle arrest through the transactivation of p21, and this pathway might inhibit cell growth and activate the apoptotic pathway by cytochrome c release and caspase activation [80-82]. Our data demonstrated that CEH induced a dose-dependent upregulation of p53 and the downstream p21, explaining the mechanism by which CEH can caused cell cycle arrest, inhibited proliferation, and induced apoptosis. In addition, p53 may regulate the sensitivity of chemotherapeutic agents as a clinical study indicated that p53 mutation may contribute to MDR [83]. Overexpression of p53 increases chemo-sensitivity in drug-resistant cells by upregulating the expression of pro-apoptotic protein p21 and Bax in osteosarcoma [84]. Upregulation of p53 specifically downregulates P-gp expression [85]. Our findings implied a role of CEH in regulating p53 and determining drug resistance of ESCC. It is clearly demonstrated that one of the mechanisms of CEH downregulation of P-gp expression is upregulation of p53, and this may result in better inhibition of ESCC growth.

Apoptosis has been linked to the formation of hetero- and homo-dimers generated via Bcl-2-Bax interactions, and it has been reported that Bcl-2 lead to cell resistance to the cytotoxic effects of a number of anticancer agents including cDDP [86]. So, we examined the Bcl-2-Bax signaling pathway and confirmed that CEH combined with cDDP could significantly downregulate the expression of Bcl-2 in Eca109 and Eca109/CDDP but did not affect Bax. This result demonstrates that CEH reversed MDR in ESCC by modulating complex signal transduction.

In the ESCC xenograft models, we also discovered that CEH increased the sensitivity of cDDP in ESCC. CEH or cDDP alone could play a role in inhibiting the growth of esophageal cancer *in vivo*, however, the treatment of combined CEH and cDDP significantly enhanced the inhibition of growth compared to the cDDP monotherapy group. The anti-tumor effect was dependent on the concentration of CEH and 10 mg/kg CEH combined with cDDP had the strongest inhibitory effect on ESCC. Similar to the *in vitro* results, CEH reversed cDDP resistance in ESCC via activation of c-Jun/JNK signaling pathway *in vivo*.

In summary, our study demonstrated that CEH could effectively reverse the MDR-mediated cisplatin resistance of ESCC cells *in vitro* and *vivo*, and was able to induce significant apoptosis in human ESCC cell lines Eca109 and its resistant strain Eca109/CDDP, inhibited ESCC cell lines proliferation and induced G2/M phase cell cycle arrest. This study demonstrated the central importance of CEH in cDDP resistance reversal in ESCC and the collective findings showed the mechanistic link between CEH and JNK/p53, indicating that CEH is a potential resistant therapeutic medicine for ESCC. It offers evidence for further research and development of this drug for cancer chemotherapy.

## MATERIALS AND METHODS

### Cell lines and cell culture

Eca109 cells were purchased from Shanghai Institutes for Biological Sciences, Chinese Academy of Sciences (Shanghai, China). Eca109/CDDP cells were derived from Eca109 (Patent No. CN201511007006.2). The two cell lines were cultured in RPMI-1640 culture medium with 10% fetal bovine serum (FBS) and 100 U/ml penicillin/streptomycin at 37 °C in a humidified atmosphere of 5% CO_2_. To maintain the resistance, 1 μg/ml of cDDP was added to Eca109/CDDP. When the cells reached confluency, they were harvested and plated for either subsequent passages or drug treatments. The trypan blue exclusion test was used throughout the experiments to check cell viability.

### Cell viability assay

Cell viability was assessed with the MTT (3-(4,5-dimethylthiazol-2-yl)-2,5-diphenyltetrazolium bromide) assay. Exponentially growing cells were plated in 96-well culture plates (∼4,000/well in 100 μl medium), cultured overnight, and incubated with a series of concentrations of CEH (0-100 μM) or cDDP (0-50 μg/ml) for 48 h. After adding 10 μl MTT solution per well, the plates were incubated at 37°C for 4 h, then the medium was removed, formazan crystals solubilized in 100 μl DMSO (dimethylsulfoxide) per well, and the absorbance (A) was measured at a wavelength of 570 nm on a microplate reader (Elx800, Biotek). The inhibition ratio was calculated as follows: (A_control_ - A_treated_)/A_control_ × 100%, where A_treated_ and A_control_ are the absorbance of the treated and control cells after 48-h incubation, respectively.

### Calculation of the combination effect index

We determined the inhibitory effects of CEH and cDDP using the MTT assay. We used the combination index (CI) described by Chou and Talalay [71, 72] for analysis and performed it by applying CalcuSyn software. CI < 1 indicates synergism; CI = 1 indicates summation; CI > 1 indicates antagonism.

### Cell cycle analysis

Cells were exposed to CEH or cDDP alone or in combination for 48 h, harvested in cold phosphate-buffered saline, fixed in 70% ethanol, stored overnight at 4 °C, and resuspended in 50 μg/ml propidium iodide (PI) staining reagent containing 100 μg/ml RNase and incubated for 30 min in the darkness. Cells were analyzed by flow cytometry (FACSCalubur, BD).

### Colony formation assay

Cells were trypsinized to single cell suspensions and seeded in 6-well plates at a density of 40,000/well. After 168 hours of culture, the colonies were stained with Giemsa solution and the clone formation ratio was counted.

### Annexin V-FITC/PI analysis

Cells were exposed to CEH or cDDP alone or in combination for 48 h, harvested in cold phosphate-buffered saline, resuspended in 500 μl incubation buffer containing Annexin V-FITC and PI, incubated in the dark for 15 min, and analyzed by flow cytometry (FACSCalubur, BD).

### Western blotting

Cell were treated with CEH and cDDP for 48 h, harvested, washed twice in ice-cold PBS, and lysed in sodium dodecyl sulfate (SDS) lysis buffer (SDS: phenylmethylsulfonyl fluoride = 50:1) at 100 °C for 20 min. Lysates were centrifuged (12,000 rpm) at 4 °C for 15 min and the supernatant was collected. Equal amount of lysate (20-30 μg) was denatured in 5× SDS sample buffer, resolved with 12% SDS-polyacrylamide gel electrophoresis, transferred to polyvinylidene difluoride membranes (Millipore), blocked with 5% skimmed milk in Tris-buffered saline containing 0.1% Tween-20 (TBST) at room temperature for 1 h, and probed with primary antibody (1:1,000) overnight at 4 °C. The membranes were incubated with secondary antibody (1:5,000) for 1 h at room temperature. Protein bands were visualized using an enhanced chemiluminescence kit (Beyotime, Shanghai, China) and imaged by autoradiography. Immunoblot was performed for p21, p53, Caspase-3, Caspase-9, Apaf-1, Cytochrome c (Cyto-c), MDR1/ABCB1 (P-gp), c-Jun, p-c-Jun, c-Jun N-terminal kinase (JNK), phospho-JNK (p-JNK), poly ADP ribose polymerase (PARP), Bcl-2, Bax, and for GAPDH as a loading control.

### Quantitative real-time RT-PCR (qRT-PCR)

Total RNA was extracted using Trizol reagent (Tiangen, Beijing, China). All RNA samples were measured by spectrophotometry and were reverse-transcribed into cDNA by using PrimerScript Master mix (Takara Biotechnology, China) according to the manufacturer’s protocol. The mRNA level was evaluated by qRT-PCR with SsoAdvanced Universal SYBR Green Supermix (Bio-Rad, Hercules, CA, USA) and was analyzed with a C1000 Thermal Cycler (CFX96 Real-Time System, Bio-Rad). Each sample was analyzed in triplicate. Relative mRNA levels were calculated using the comparative threshold cycle (CT) with the analyzed gene expression levels normalized by those of GAPDH. Forty cycles (95 °C for 3 min, 95 °C for 5 s, 59 °C for 5 s) were performed on the Light Cycler in a 10 μl reaction volume, followed by the generation of a melting curve. The relative changes in gene expression were calculated with the 2^-ΔΔCt^ method, where ΔΔCt = ΔCt (drug treated) - ΔCt (control) for RNA samples.

Gene-specific primer pairs used in this study were as follows: *MDR1* Forward: 5’-CTGCTTGATGGCAAAGAAATAAAG-3’, *MDR1* Reverse: 5’-GGCTGTTGTCTCCATAGGCAAT-3’; *GAPDH* Forward: 5’-GAGTCAACGGATTTGGTCGT-3’, *GAPDH* Reverse: 5’-GACAAGCTTCCCGTTCTCAG-3’.

### Xenograft tumor assay in nude mice

BALB/c-nu/nu nude female mice (4–6 weeks) were purchased from the Institute of Laboratory Animal Sciences, Chinese Academy of Medical Sciences in Beijing, China; and housed in a specific pathogen-free (SPF) environment. Eca109 (3 × 10^6^) and Eca109/CDDP cells were injected subcutaneously (s.c.) into the flanks of mice. When tumors grew to ∼ 6 mm in diameter, mice were grouped into eight groups (five mice per group). The treatment protocol with various concentrations of CEH or cDDP *in vivo* is shown in Figure 6A. The eight groups were treated with vehicle control or CEH through intra-peritoneal (i.p.) injection every day, cDDP was administered once every 5 days. The volume of administration was 10 μl/g. Tumor volumes were measured at the start of the treatment and every 2 days during the course of the therapy. The tumor length (L) and width (W) were measured, and the tumor volume (V) was calculated as follows: V = ½ × L × W^2^. Tumors were resected on the second day following the last injection, and weighed. The percentafe of tumor growth inhibition was calculated by comparing the tumor weight average values of the treated groups with those of the tumor-bearing control group. Tumor growth in saline treated control animals was taken to be at 100%. Tumors tissues from every mouse were homogenized and lysed for western blot analysis. The remaining tissues were embedded in paraffin, sectioned at 3 μm, and stained with hematoxylin and eosin (H&E). All experiments were performed in accordance with national ethical guidelines and with the approval from the Institutional Animal Care and Use Committee of Jinan University.

### H&E-staining, immunohistochemistry (IHC) & immunofluorescence (IF)

For H&E, IHC, and IF analysis of the ESCC xenograft tumors, tissue sections were cut at 3 μm. Sections were deparaffinized using xylene and then rehydrated with a graded alcohol series and finally distilled water. After being treated with 3% H_2_O_2_ for 15 min, the slides were treated for antigen retrieval in 121 °C for 5 min, and then slowly cooled down to room temperature. The slides were put into the hematoxylin solution for several minutes. After being separated in acid water and ammonia water, slides were dehydrated for 10 minutes with 70% and 90% alcohol and stained with eosin, the stained sections were dehydrated in pure alcohol and cleared with xylene. After a 30 min incubation in 10% goat serum, the sections were incubated overnight with proper primary antibodies (1:1,000 dilution). After being washed three times with PBS, the sections were incubated with HRP-conjugated secondary antibodies (1:500 dilution), and the subsequent detection was performed using the standard substrate detection of HRP. 3,3′-diaminobenzidine (DAB) development and hematoxylin and eosin staining were performed according to standard protocols. The slides were observed under a fluorescence microscope (Eclipse Ti, Nikon) and photographed.

### Statistical analysis

Data are represented as means ± SD from triplicate samples of at least three independent experiments. Differences between the mean values were analyzed by two-sample Student’s *t*-test and one-way analysis of variance; p-values below 0.05 were considered to be statistically significant.

## SUPPLEMENTARY MATERIALS FIGURE


