# Correction: Cepharanthine hydrochloride reverses the mdr1 (P-glycoprotein)-mediated esophageal squamous cell carcinoma cell cisplatin resistance through JNK and p53 signals

**DOI:** 10.18632/oncotarget.27638

**Published:** 2021-01-05

**Authors:** Pengjun Zhou, Rong Zhang, Ying Wang, Dandan Xu, Li Zhang, Jinhong Qin, Guifeng Su, Yue Feng, Hongce Chen, Siyuan You, Wen Rui, Huizhong Liu, Suhong Chen, Hongyuan Chen, Yifei Wang

**Affiliations:** ^1^ Guangzhou Jinan Biomedicine Research and Development Center, Guangdong Provincial Key Laboratory of Bioengineering Medicine, College of Life Science and Technology, Jinan University, Guangzhou 510632, Guangdong, P. R. China; ^2^ Department of Pathogen Biology and Immunology, School of Basic Course, Guangdong Pharmaceutical University, Guangzhou 510006, Guangdong, P. R. China; ^3^ State Key Laboratory of Oncology in South China and Collaborative Innovation Center for Cancer Medicine, Sun Yat-sen University Cancer Center, Guangzhou 510060, Guangdong, P. R. China; ^4^ Guangdong Food and Drug Vocational College, Guangzhou 510520, Guangdong, P. R. China; ^5^ Guangzhou Institute of Pediatrics, Guangzhou Women and Children’s Medical Center, Guangzhou Medical University, Guangzhou 510623, Guangdong, P. R. China; ^6^ Department of Hepatobiliary Surgery, Xijing Hospital, Fourth Military Medical University, Xi’an 710032, Shanxi, P. R. China; ^7^ Guangdong Provincial Engineering Center of Topical Precise Drug Delivery System, Guangdong Pharmaceutical University, Guangzhou 510006, Guangdong, P. R. China

**This article has been corrected:** Due to errors during image assembly, the images for Figure 2, panels E and F, were accidentally switched. The corrected Figure 2 is shown below. The authors declare that these corrections do not change the results or conclusions of this paper.

Original article: Oncotarget. 2017; 8:111144–111160. 111144-111160. https://doi.org/10.18632/oncotarget.22676

**Figure 2:**
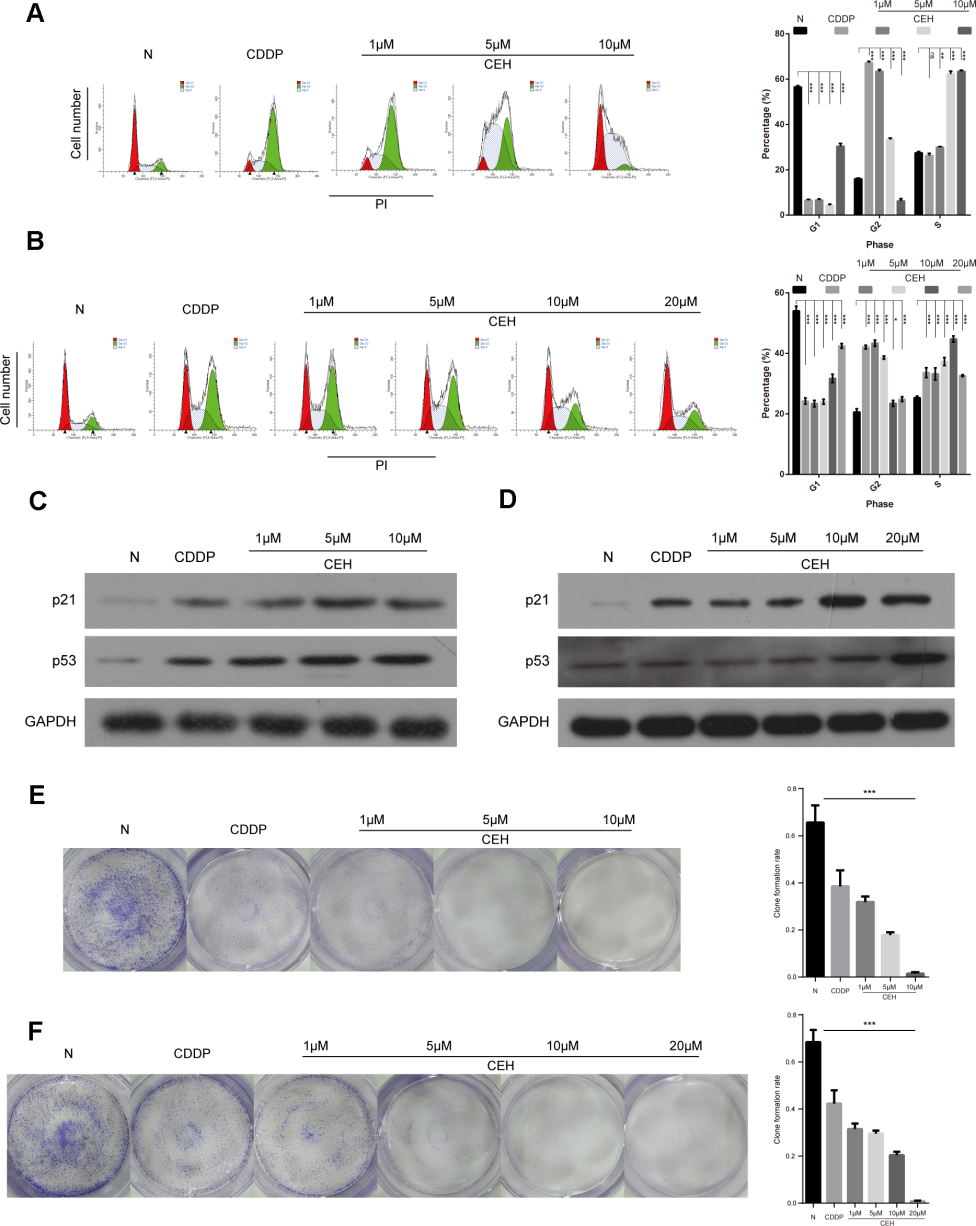
Induction of cell cycle arrest and inhibition of cell proliferation by cisplatin (cDDP) alone and combined with cepharanthine hydrochloride (CEH) in esophageal cancer cell lines. (**A** and **B**) Cell cycle analysis. Percentages of Eca109 and Eca109/CDDP cells in the G1, S, and G2/M phases are presented respectively. Effects of cDDP and combined with various concentrations of CEH medication on cell cycle distribution. Eca109 (A) and Eca109/CDDP (B) cells were treated with 0, 1, 5, 10 and 20 μM CEH combined with cDDP for 48 h, and cell cycle distribution was measured by flow cytometry after PI staining. (**C** and **D**) p21 and p53 protein levels were determined by western blot analyses. GAPDH was used as the loading control. (**E** and **F**) Cells were treated with 0, 1, 5, 10 and 20 μM CEH combined with cDDP for 48 h; representative images of Eca109 (E) and Eca109/CDDP (F) clone formation are shown. ***, P < 0.001 compared with the control.

