# Supplementary material for: Cepharanthine hydrochloride reverses the mdr1 (P-glycoprotein)-mediated esophageal squamous cell carcinoma cell cisplatin resistance through JNK and p53 signals
Source: Oncotarget. 2017 Nov 27;8(67):111144–60. doi: 10.18632/oncotarget.22676 (PMC5762312; doi:10.18632/oncotarget.22676)
Supplement: Supplementary file 1 [file oncotarget-08-111144-s001.pdf]

Cepharanthine hydrochloride reverses the mdr1 (P-glycoprotein)-mediated esophageal squamous cell carcinoma cell cisplatin resistance through JNK and p53 signals

SUPPLEMENTARY MATERIALS

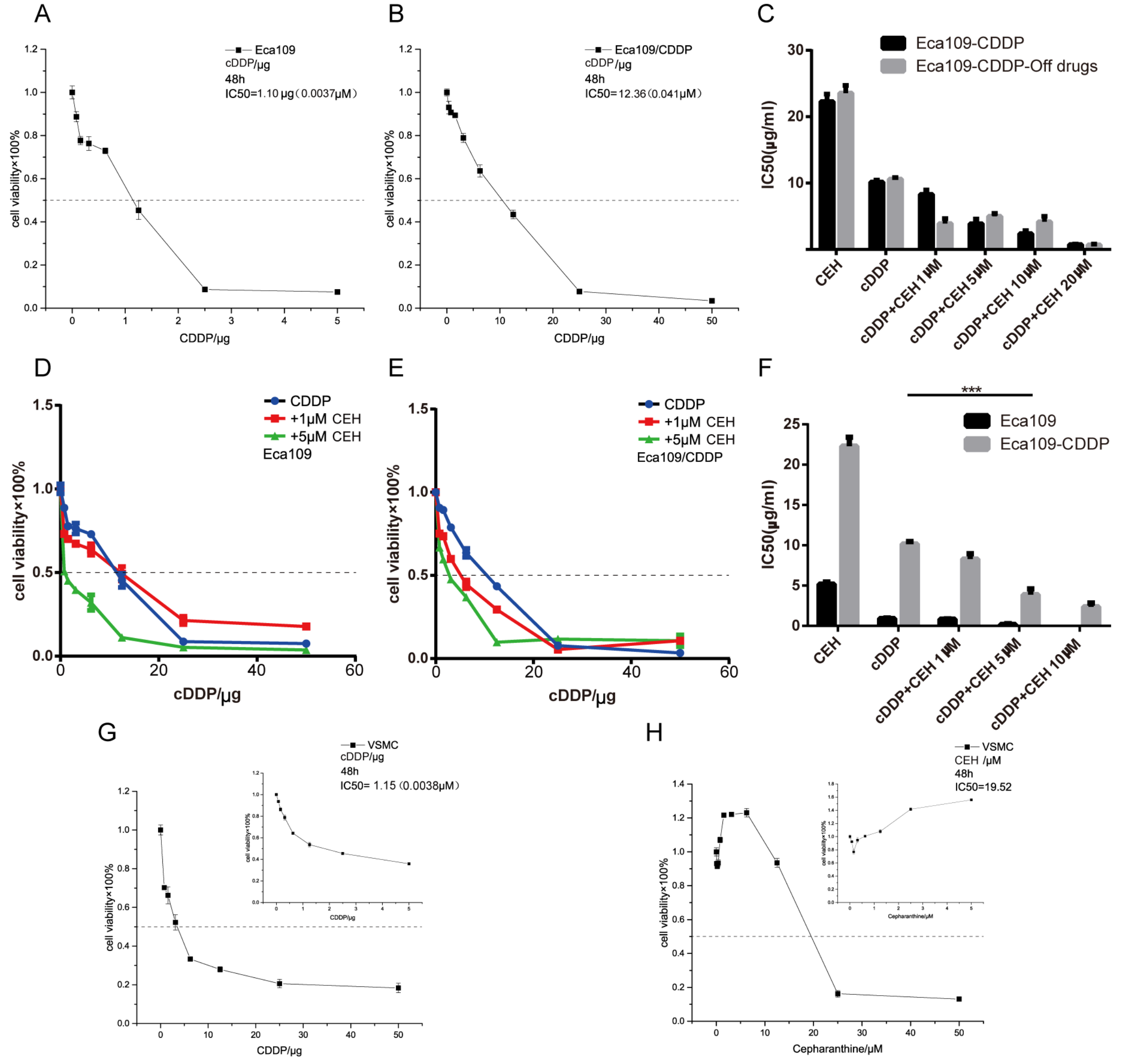

Supplementary Figure 1: (A and B) Effects of cisplatin (cDDP) on the growth of the esophageal squamous cell carcinoma cell lines, Eca109 and Eca109/CDDP. Cells were treated with various concentrations of cDDP for 48 h and cell viability was measured by MTT assay. **(C)** Effect of drug withdrawal on resistance of the resistant cell lines. **(D to F)** The effects of 48 h treatment with cDDP and cDDP combined with various concentrations of cepharanthine hydrochloride (CEH) on the growth of Eca109 and Eca109/CDDP cells (D and E). The half maximal inhibitory concentration (IC50) was quantified (F). **(G and H)** Effects of CHE and cDDP on the growth of the human aortic smooth muscle cells HA-VSMC. Cells were treated with various concentrations of CEH or cDDP for 48 h, and cell viability was measured by MTT assay. All data are presented as the mean ± SD of three independent experiments. **, $P < 0.01$; ***, $P < 0.001$ compared to the control.
